# Blocking HTLV-1/2 silent transmission in Brazil: Current public health policies and proposal for additional strategies

**DOI:** 10.1371/journal.pntd.0009717

**Published:** 2021-09-23

**Authors:** Carolina Rosadas, Maria Luiza B. Menezes, Bernardo Galvão-Castro, Tatiane Assone, Angélica E. Miranda, Mayra G. Aragón, Adele Caterino-de-Araujo, Graham P. Taylor, Ricardo Ishak

**Affiliations:** 1 Section of Virology, Department of Infectious Disease, Imperial College London, London, United Kingdom; 2 Departamento Materno-Infantil, Faculdade de Ciências Médicas, Universidade de Pernambuco, Pernambuco, Brazil; 3 Centro Integrativo e Muldisciplinar de Atendimento ao Portador de HTLV (CHTLV), Escola Bahiana de Medicina e Saúde Pública, Salvador, Bahia, Brazil; 4 Faculdade de Medicina, Instituto de Medicina Tropical de São Paulo, Universidade de São Paulo, São Paulo, Brazil; 5 Programa de Pós-Graduação em Doenças Infecciosas, Universidade Federal do Espírito Santo, Espírito Santo, Brazil; 6 Laboratório de Pesquisa em HTLV, Centro de Imunologia, Instituto Adolfo Lutz, São Paulo, Brazil; 7 Laboratório de Virologia, Instituto de Ciências Biológicas, Universidade Federal do Pará, Pará, Brazil; Beijing Children’s Hospital, Capital Medical University, CHINA

## Abstract

Human T-cell lymphotropic viruses 1 and 2 (HTLV-1/2) are relatively common in Brazil but remain silent and neglected infections. HTLV-1 is associated with a range of diseases with high morbidity and mortality. There is no curative treatment for this lifelong infection, so measures to prevent transmission are essential. This narrative review discusses HTLV-1/2 transmission routes and measures to prevent its continuous dissemination. The public health policies that are currently implemented in Brazil to avoid HTLV-1/2 transmission are addressed, and further strategies are proposed.

## Introduction

Human T-cell lymphotropic virus 1 and 2 (HTLV-1/2) are retroviruses that cause persistent infection, mainly in T-cell lymphocytes. HTLV-1 is associated with high morbidity and mortality clinical conditions, while HTLV-2 is rarely associated with disease. Transmission occurs through sexual relations, transfusion/transplantation of blood/organs infected with the virus, and from mother to child, mainly by breastfeeding. Although it has a worldwide distribution, except for Japan, low- and middle-income countries and predominantly ethnic minority population groups in high-income nations are the most affected. It has been estimated that 800,000 to 2.5 million people are living with HTLV-1 (PLHTLV) in Brazil [1,2].

A large proportion of infected persons will remain asymptomatic throughout their entire life, but many PLHTLV will develop one or more of a range of diseases. HTLV-1–associated myelopathy (HAM) is a debilitating condition that results from an inflammatory response mainly within the spinal cord, but with increasing evidence of widespread brain inflammation. HAM greatly impacts patient’s autonomy and quality of life, and, during follow-up, about 50% become wheelchair dependent [3–6]. In Brazil, the risk of HAM and other neurological diseases is reported to be higher than usual at 5% [7–9]. In addition to the high morbidity, HAM was also identified as a significant predictor of increased mortality in PLHTLV from Brazil (hazard ratio (HR) 5.03, 95% CI 1.96 to 12.91) [10]. Adult T-cell leukaemia (ATL) is an aggressive neoplasm caused by HTLV-1 with a median survival of 8 months and affects about 4% of carriers [11]. In Brazil, ATL is probably underdiagnosed [12]. Other inflammatory diseases, including uveitis, keratoconjunctivitis, infective dermatitis, arthritis, and a range of pulmonary pathologies may also occur as a consequence of HTLV-1 infection. Moreover, the impact of HTLV-1 on several coinfections, including *Strongyloides stercoralis* and *Mycobacterium tuberculosis* is recognised [13,14]. Indeed, HTLV-1 infection is associated with an increased risk of all-cause mortality (adj MR 1.57) not explained by the two well-characterised HTLV-1–associated diseases, ATL and HAM [13].

Asymptomatic and subclinical HTLV-1/2 infections facilitate the maintenance and spread of HTLV-1/2 within communities. The long incubation period in many cases of HTLV-1–associated diseases, which usually occur in late adulthood, and the underdiagnosis of infection increase the risk of HTLV-1/2 silent transmission. There is no vaccine or curative treatment for HTLV-1/2. Available antiretroviral therapy has no beneficial effect on established HTLV-1/2 infection, and it is not medically indicated [15]. Therapeutic intervention, however, should focus on the management of clinical diseases associated with the infection and to improve patients’ autonomy and quality of life [15–20]. A multidisciplinary approach towards PLHTLV care is recommended to optimise care and outcomes [21].

HTLV-1/2 are neglected infections, but prevention of transmission is an attainable goal. HTLV-1/2 are cell-associated viruses with provirus present in the blood, semen, vaginal fluid, and maternal milk, and every effort should be directed to eliminate transmission related to these fluids. Following a public call for action to eradicate the virus [22,23], the World Health Organization (WHO) conducted a global consultation on HTLV-1, aiming to review existing data to better understand the public health implications of HTLV-1 and its associated diseases. Public health measures currently implemented in different countries were presented, and the report identified that Japan alone has an overall national programme for HTLV-1 infection [24]. There is limited information about the policies available in Brazil, one of the countries most affected by both HTLV-1 and HTLV-2. Although there is no formal Brazilian HTLV-1/2 national programme yet, there are several policies dating back as far as 1993, and, Bahia, the state with the highest prevalence of HTLV-1 infection in the country, established a protocol for the integrated care of PLHTLV at the end of 2020 [25]. Here, we discuss the public health policies that are already implemented in Brazil and propose further strategies to detect and prevent HTLV-1/2 spread in the country.

## Methods

In addition to the policy documents known to the authors, the public website of the Brazilian Ministry of Health (MoH) was interrogated for relevant policies using the search term “HTLV” in each of their clinical guidelines and therapeutic protocols. Each policy was then examined to confirm the applicability to HTLV infection. All relevant policies were then included in the narrative following additional strategies discussed by the authors and recommendations agreed.

### Policies that are implemented in Brazil that may impact HTLV-1/2 transmission

#### HTLV-1/2 as a sexually transmitted infection (STI)

HTLV-1/2 infected cells are present in seminal and vaginal fluid and are transmitted by unprotected sexual intercourse [26–29]. High HTLV-1 DNA copy number (also known as proviral load, PVL) in circulating peripheral blood mononuclear cells (PBMCs) is a known risk factor for sexual transmission, and the efficiency of HTLV-1 transmission is higher from male to female than from female to male [26]. Some authors hypothesise that penile ulcers and lesions on vaginal mucosa, which may occur as a result of other sexually transmitted infections (STIs), may facilitate HTLV-1 sexual transmission. In fact, cervicitis increases HTLV-1 shedding, and the prevalence of HTLV-1 infection is higher in patients with STI, such as syphilis, chlamydia, and human papilloma virus [30,31].

Effective prevention of HTLV-1/2 sexual transmission is primarily linked to the use of barrier methods during sexual activity. However, public policies to prevent this route of infection should not rely solely on safer sex, but rather on an integrated strategy, requiring biomedical, behavioural, and structural interventions, as for other STI [32]. Perversely, strategies developed to prevent HIV transmission may result in increased exposure to HTLV if reliance on, for example, HIV-specific pre-exposure prophylaxis (PreP) displaces broader STI reduction approaches, unless antiretrovirals that inhibit both viruses (particularly integrase inhibitors) are used.

Screening for HTLV-1 is compulsory for both donors and recipients for assisted reproduction. HTLV-1 infection is considered an exclusion criterion for reproductive gamete donors since 2011 [33]. It is important to stress that HTLV-1 infection should not be an obstacle for HTLV-1 discordant couples wishing to procreate. Medical advice should be sought, and couples should be tested for other STI. If the woman is infected by HTLV-1, assisted reproduction (domestic insemination) should be offered. If the man is infected, sperm washing followed by artificial insemination could be considered based on biological principle. This process, current in use for HIV discordant couples, should be highly effective in preventing HTLV-1 transmission by separating the sperm from the seminal fluid and cells. This is recommended by the American Society for Reproductive Medicine [34]. However, the European Society of Human Reproduction and Embryology (ESHRE) concluded that there is not enough evidence to recommend the routine use of advanced processing of semen from HTLV-1/2 seropositive patients [35]. In the absence of sperm washing, the use of barrier contraception except following ovulation with support and education for the couple to identify the fertile period could be considered as a risk reduction strategy. It is worth noting that published transmission data are focused on discordant couples where HTLV transmission has not occurred despite prior unprotected sexual intercourse and that the risk of transmission in new relationships may be considerably higher. A case of HAM following a single sexual exposure to HTLV-1 has been reported [36].

HTLV-1 is already included in the Clinical Protocol and Therapeutic Guidelines for Sexually Transmitted Infections (PCDT IST) published by the Brazilian MoH [32]. Information about HTLV-1 is also available on the website of the Department of Chronic Diseases and Sexually Transmitted Infections of MoH. Additional strategies aiming to prevent HTLV-1/2 sexual transmission should include the following:

1. screening the sexual contacts of PLHTLV;
2. counselling of PLHTLV to assure that they are aware of the chronic aspect of HTLV-1 infection, its transmission routes, and how to prevent new infections;
3. HTLV-1 screening in people with other STI; and
4. increasing awareness about HTLV-1 in the general population, among healthcare workers and ensuring that HTLV-1/2 is included in STI awareness campaigns.

#### HTLV-1/2 as a blood-borne infection

HTLV-1/2 screening of blood donors was implemented in Brazil in 1993 and constitutes a key measure to control parenteral transmission of these viruses [37]. Donors and recipients of organs, tissues, cells, or body parts are also screened since 2009 [38]. HTLV-1/2 infection is a definitive exclusion criterion. ATL and HAM has been described in patients infected peri-transplantation. The risk of HTLV-1 infection following solid organ transplantation is 100%, and the risk of HAM following HTLV-1 infection acquired by this route is high (40%) and occurs after a relatively short interval (3.8 years) [39–41]. The risk acceptance of HTLV-1 infection as a consequence of solid organ transplantation was investigated in Canada and confirmed that patients would not be willing to forego HTLV-1 screening of solid organs donors [42].

The current Brazilian policies to prevent HTLV-1/2 parenteral transmission are important but still need improvement. A crucial point is to include complementary tests (confirmatory and discriminatory for HTLV-1 and HTLV-2) for those persons with seroreactive tests in the screening assay. Positive ELISA tests should be followed by western blot (WB) and/or PCR to avoid false-positive results and the uncertainty and discomfort that unconfirmed reactivity generates for the donors. Assays for confirmatory testing for HTLV-1/2 were made available to the public health system in 2016 but are limited to those persons who need laboratory confirmation of ATL [43]. The inclusion of line immunoassay (LIA) is recommended based on its superior performance compared to WB [44–46]. A screening test is insufficient to diagnose HTLV-1/2 infection, and confirmation is necessary for those who are willing to donate their blood and organs. Blood and organ donation is, above all, an act of kindness and selflessness. It is not unusual to come across individuals who have been informed of a reactive HTLV-1/2 ELISA test with no further testing, who are subsequently shown to be uninfected, sometimes years after being informed of the screening test result. The impact of a false-positive HTLV-1/2 diagnosis is profound, influencing key life decisions and should not be underestimated. Using the methodology previously described [47] and considering (1) 3.3 million of blood donors; (2) specificity of screening test [48]; and (3) prevalence of HTLV-1 infection in blood donors [2], we have estimated that HTLV-1/2 screening would result in 8,580 to 256,080 false-positive results annually. The lack of national reference centres for counselling and follow-up of PLHTLV or those with seroreactivity in screening test adds uncertainty for those newly diagnosed individuals.

Needle sharing among people who inject recreational drugs is another important route of HTLV-1/2 parenteral dissemination. High prevalence rates of HTLV-1/2 among this specific population have been frequently reported [2,49–51]. A comprehensive strategy focusing on this vulnerable population is of utmost importance. These are already undergoing as part of HIV-1 and other STI prevention programmes and comprise a range of activities including more simple strategies such as offering harm reduction initiatives, provision of sterile needles for people who inject drugs, and enabling the safe disposal of needles and syringes up to more complex interventions. Complex interventions comprise behavioural and structural changes aiming to reduce stigma, inequalities, and any barrier that may prevent access to universal health. HTLV-1/2 transmission by skin scarification and self-flagellation has been reported in indigenous people and during religious ceremonies [52–54].

#### HTLV-1/2 mother-to-child transmission

HTLV-1/2 is transmitted from mother to child, mainly by breastfeeding. Residual transmission may occur in about 2.5% of those children that are exclusively fed by milk formula substitutes, indicating that transmission may occur during pregnancy or delivery [55]. In Brazil, in urban areas, mother-to-child transmission rate was reported to be 14.1% [56], with an estimated number of new infections reaching at least 16,500 new cases annually [57]. Low-income pregnant women with (i) high HTLV-1 PVL in blood and milk; (ii) breastfeeding for longer periods; (iii) HLA concordance between mother and child; (iv) *Strongyloides* sp. coinfection; and (v) patients with HAM have a higher risk of transmitting HTLV-1 to their children [55]. Vertical transmission of HTLV-1 is not only implicated with higher risk of adverse clinical outcome associated with HTLV-1 infection but also with maternal feelings of guilt [58,59]. Among highly vulnerable indigenous communities in the Amazon region of Brazil, mother-to-child transmission of HTLV-2 is common and reaches as high as 30% [54,60]. There are no clear consequences of the perpetuation of this virus among semi-closed and isolated communities nor policies towards the elimination of HTLV-2 among these population groups.

The Brazilian MoH recommends the interruption of breastfeeding, using pharmaceutical intervention of mothers to suppress lactation (Cabergolin), and provision of milk formula substitutes for those babies born of HTLV-1/2 seropositive women [32,61]. The first step should be to identify those pregnant women that are infected with HTLV-1/2. A national antenatal screening programme is of utmost importance and should be a priority in the country. Some Brazilian States, including Bahia, Minas Gerais, and Mato Grosso do Sul, have implemented HTLV-1/2 policies for antenatal screening, but the measure should be expanded and implemented nationally, as in Japan. In fact, Japan first implemented regional antenatal screening combined with other policies to avoid mother-to-child transmission in the late 1980s [62,63]. The national screening programme, established in 2010, is one of the main pillars to achieve their aim to eliminate HTLV-1/2 infection in Japan. It is worth mentioning that short-term breastfeeding and use of freeze and thaw maternal milk were reported to reduce the risk of transmission but not as effectively as the avoidance of breastfeeding. Although all the measures were initially recommended in Japan, this was revised in 2016 to recommend exclusive milk formula substitutes feeding for the babies of all mothers who are positive for HTLV-1 [63]. In situations where exclusive formula feeding is not acceptable, feasible, affordable, sustainable, and safe (AFASS), alternative methods to reduce the risk of transmission may still be considered. Limiting the duration of breastfeeding is not always practical or achieved, and, therefore, in such settings, innovative alternatives are needed.

**Fig 1:**
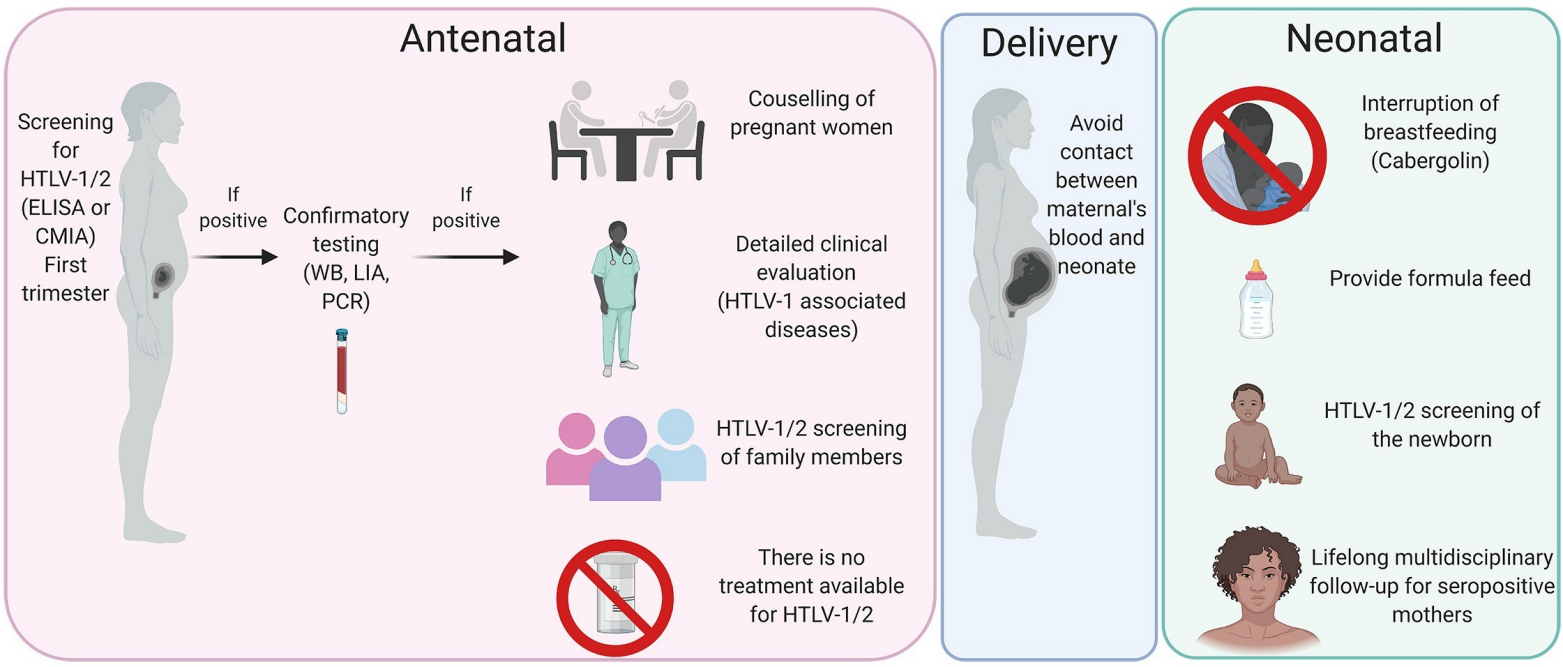
Flowchart proposal for management of HTLV-1/2 infection in pregnant women and prevention of mother-to-child transmission. CMIA, chemiluminescence assay; HTLV-1/2, human T-cell lymphotropic virus 1 and 2; LIA, line immunoassay; WB, western blot.

A graphic summary for the proposed clinical management of HTLV-1/2 infection in pregnant women and prevention of mother-to-child transmission is shown in Fig 1. Antenatal screening is strongly recommended at the first trimester, which would allow proper confirmatory testing and counselling for those seropositive mothers prior to delivery. The diagnostic algorithm should be similar as to the general population, using an ELISA or CMIA as screening test, followed by confirmation using PCR, WB, or LIA [64]. Pregnancy does not adversely affect HTLV-1 diagnosis[65]. Counselling and detailed clinical evaluation should be offered to identify signs or symptoms of any HTLV-1/2–associated diseases, and it is important to reinforce the information about the risk of HTLV-1/2 transmission and importance of avoidance of breastfeeding. Stigma and prejudice may impair the adherence of new mothers to the recommendations [66,67]. A recent study commissioned by WHO identified that women infected by HTLV express concerns about social perceptions of not breastfeeding, as well as the artificial alternatives of breast milk[59,66]. Mothers commonly indicate that they are afraid of not bonding to the child if they do not breastfeed [59]. Misconceptions must be fully discussed during counselling. In addition, the limited knowledge of healthcare professionals about HTLV-1/2 should be addressed in order to avoid misleading recommendations [68].

HTLV-1/2 testing should be offered for relatives, including sexual partners and children of previous pregnancies. Antiretroviral drugs do not decrease HTLV-1/2 PVL in established infection and are not currently recommended for the prevention of mother-to-child transmission, although this has been offered in very high–risk cases of mothers with ATL in pregnancy [69]. Although some argue that cesarean section should be recommended to pregnant women with HTLV-1/2 infection [70], there is no consensus about the impact of the procedure in reducing the risk of HTLV-1/2 mother-to-child transmission. However, avoiding contact between maternal blood and the neonate during delivery was linked to the reduction of HTLV-1 transmission among exclusive formula-feed children in Japan [71]. In Brazil, when a woman living with HTLV-1/2 is submitted to cesarean section, immediate clamping of the umbilical cord is recommended [72].

Postnatal care should include early cessation of breastfeeding. Milk formula substitutes should be offered for women living with HTLV-1/2, which is already available in some Brazilian states, including Bahia and São Paulo. Screening of infants is necessary to rule out mother-to-child transmission of HTLV-1/2, and the aforementioned diagnostic algorithm can be used if the child is older than 18 months. If the child is tested earlier, maternal antibodies may be detected by serological assays. Seroconversion has been shown in long-term follow-up studies with incident infection in breastfed babies as late as 3 years of age [73,74]. No seroconversions were reported in a longitudinal study of breastfed children from age 2 to 12 years old [75]. Multidisciplinary lifelong follow-up is needed for PLHTLV. Specialised centres play an important role advising mothers-to-be who live with HTLV-1/2 infection. Studies showed that they are vital as the women tend to trust their advice even when faced with contradictory views from other health staff [66,68,76].

The Brazilian MoH has implemented one of the most successful antenatal programmes to prevent other STI, including HIV-1 and in three Brazilian cities (São Paulo, Curitiba e Umuarama), the elimination of vertical transmission of HIV-1 has been highly successful. The network and knowledge are already established in the country, and the prevention of HTLV-1/2 transmission should be considered as an extension of a successful existing programme. Recently, HTLV-1/2 infection has been addressed in the Clinical Protocol and Therapeutic Guidelines for Prevention of Vertical Transmission of HIV, Syphilis, and Viral Hepatitis, published by the Brazilian MoH [61].

## A neglected virus affecting a neglected population needs an integrated strategy to prevent its spread

Brazil has the highest number of PLHTLV in the world. However, within the country, HTLV-1/2 infection is not equally distributed [2]. Compulsory notification of HTLV-1/2 infection would facilitate the identification of the real situation of these viruses in Brazil. Very few countries maintain the registry of PLHTLV such as Japan and the United Kingdom [63,77,78].

Available data on HTLV-1 infection show that black/mixed ethnicities, with low income and education level, are those more frequently infected [2]. Women are mostly affected, are more susceptible to sexual transmission, and have greater risk of HAM [22,79]. In addition, they carry the burden of transmitting the virus to their children. Women who are victims of domestic and/or sexual violence have 9 times more risk of being infected by HTLV-1/2 in Brazil [80], which reinforces the observation that this neglected virus will predominantly affect vulnerable individuals. Other vulnerable population groups that are considered high risk for acquiring infection were already identified and include men who have sex with other men (MSM), commercial sex workers, and people who inject drugs. High prevalence of HTLV-1/2 infection is well documented in Brazilian indigenous populations [81,82]. Due to its complexity, HTLV-1/2 prevention needs a combined approach. Structural interventions, targeting sociocultural factors that will impact the vulnerability of individuals and specific social groups, are necessary.

## Perspective of actions

Table 1 shows the public health policies that are currently available in Brazil and the additional proposed strategies that aim to reduce HTLV-1/2 transmission. Recently, the Brazilian MoH commissioned the construction of a specific Clinical Protocol and Therapeutic Guidelines dedicated for HTLV-1/2 by a technical advisory committee, including patient representatives. This document, which is already considered a milestone in public health policies to prevent HTLV-1/2 in the country, is presently under scrutiny by the technical staff of the MoH. In Brazil, as in other parts of the world, HTLV-1/2 infection has been considered “neglected,” “silent,” or “invisible,” despite up to 10 million of PLHTLV who are mostly still unaware of their condition. A definitive step to start fighting against this deplorable situation would be to increase HTLV-1/2 awareness and to allow PLHTLV to have access to the existing public health services. Several infectious diseases have similar incidence and prevalence to HTLV-1/2, and those patients, unlike PLHTLV, have their right to receive proper diagnosis, treatment, and well-recognised support. Curiously, in Brazil, the number of HTLV-1 infected pregnant women is double that of HIV-1 (16,548 versus 8,699, respectively, in 2018) [57]; furthermore, screening for rare diseases among neonates is routine, aiming to improve the early clinical management of these patients, increase their survival, and improve their quality of life. These achievements that conquered other diseases are similar to what is needed for those infected by HTLV-1/2 and is granted by the Brazilian Constitution.

**Table 1:** Current public health policies towards HTLV-1/2 in Brazil and additional proposed interventions.

Current public policies in Brazil	Proposed interventions
**Parenteral transmission**	
Screening of blood donors (1993)	Confirmatory testing for those seroreactive donors in screening assays
Screening of organ donors and receptors (2009)	
Strategies for harm reduction to people who inject drugs*	
**Sexual transmission**	
Inclusion of HTLV-1/2 in the Clinical protocol and Therapeutic Guidelines for STI* (2020)	Provide HTLV-1 testing for sexual contacts of PLHTLV
Screening for HTLV-1 in recipients and donors for assisted reproduction (2011)	Inclusion of HTLV-1 testing in the routine care of patients with STI
HTLV-1/2 infection as an exclusion criterion for reproduction gametes donors (2011)	Offer assisted reproduction for discordant couples
Inclusion of HTLV-1 in awareness campaign for STI*	
Structural and behavioural interventions targeting the reduction of transmission of STI*	
**Mother-to-child transmission**	
Recommendation to avoid breastfeeding for HTLV-1/2 seropositive mothers (2019)	Implementation of national HTLV-1 antenatal screening programme with confirmatory testing
Provision of formula milk to seropositive mothers*	Provide counselling for HTLV seropositive pregnant women
Inclusion of HTLV-1/2 in the Clinical Protocol and Therapeutic Guidelines for Vertical Transmission*	Provide testing for family members
	Provide testing for the newborn
**Overall**	
Guideline for Clinical Management for HTLV-1/2 infection (2004 and 2013)	Compulsory notification of HTLV-1/2 infection
Research funding for HTLV-1/2*	Establishment of reference centres for the multidisciplinary follow-up of PLHTLV
Include HTLV-1 in awareness campaign for general population and healthcare workers*	Counselling of PLHTLV
Include HTLV in the STI E-learning training for Latin America	Inclusion of LIA as a confirmatory test for HTLV-1/2 infection
	Inclusion of CMIA as a screening test for HTLV-1/2 infection

* Policies that are currently implemented but need improvement and/or expansion.

CMIA, chemiluminescence assay; HTLV-1/2, human T-cell lymphotropic virus 1 and 2; LIA, line immunoassay; PLHTLV, people living with HTLV; STI, sexually transmitted infection.

Since the 1980s, there are several relevant landmarks in the country to provide information to the general laymen, to PLHTLV, or to healthcare workers. These include, but are not limited to, the publication of the Guideline for Clinical Management of HTLV-1 Infection (published in 2004 and revised in 2013), the establishment of 23rd March as the HTLV National Awareness Day, participation of representatives of MoH in scientific events that occurs every 2 years in the country, distribution of HTLV-1/2 diagnostic algorithm to primary healthcare units, and publication of a commissioned review about HTLV-1/2 infection prepared by technical advisory group. However, there is still a need for more aggressive campaigns to raise awareness of the virus. There is no compulsory notification for the HTLV-1–associated diseases, and the absence of reliable numbers posed difficulties to implement public health policies to eliminate HTLV-1/2. A national registry for pregnant women infected by HTLV-1/2 is under development. There are an estimated 2,500,000 PLHTLV in Brazil, including men, women, children, living in urban, nonurban, and specific vulnerable population groups such as pregnant women, MSM, male and female sex workers, people who inject recreational drugs, quilombolas (slave escapees), and indigenous communities. The Brazilian MoH granted research funding to better understand the prevalence and distribution of these viruses in the country. A national prevalence study on pregnant women is planned but was delayed due to the Severe Acute Respiratory Syndrome Coronavirus 2 (SARS-CoV-2) pandemic. The MoH also published recently an epidemiological bulletin about HTLV-1/2 prevalence and distribution in Brazil [2].

Brazil is a large country with a well-established healthcare system that is able to assist patients, to provide antenatal care to pregnant women, has an integrative conglomerate of centres for blood donors, and the ability to provide synchronous mass administration of vaccines. The inclusion of the proposed strategies to prevent HTLV-1/2 in this network requires just a discrete financial and administrative adaptation but is crucial to the benefit of the general population and to the Brazilian citizen living with HTLV-1/2.

**Key learning points**

- Human T-cell lymphotropic virus (HTLV) is associated with high morbidity and mortality diseases, and there is no curative treatment available for this infection.
- HTLV is an important health problem in Brazil, and the country has implemented several HTLV public health policies, dating back to 1993, and is continuously advancing in establishing measures to control the virus.
- Public health policies include universal screening of organs, tissues, and reproductive gametes recipients and donors and blood donors; recommendations on avoidance of breastfeeding for seropositive mothers; and a national programme for sexually transmitted infection (STI) with HTLV as a priority.
- HTLV is a neglected virus, causing neglected diseases affecting vulnerable populations; thus, an integral approach to prevent its transmission is needed.
- Proposed public health policies comprise universal antenatal screening, inclusion of confirmatory testing for those reactive in screening assays, compulsory notification, establishment of multidisciplinary reference centres, offer of testing to high-risk population, and public education to increase awareness.

**Top five papers**

1. WHO. Human T-lymphotropic virus type 1: technical report. [Internet]. 2021. Available from: https://www.who.int/publications/i/item/9789240020221
2. Gessain A, Cassar O. Epidemiological Aspects and World Distribution of HTLV-1 Infection. Front Microbiol. 2012;3:388. 10.3389/fmicb.2012.00388
3. Martin F, Tagaya Y, Gallo R. Time to eradicate HTLV-1: an open letter to WHO. Lancet. 2018;391:1893–4. 10.1016/S0140-6736(18)30974-7
4. Hino S. Establishment of the milk-borne transmission as a key factor for the peculiar endemicity of human T-lymphotropic virus type 1 (HTLV-1): the ATL Prevention Program Nagasaki. Proc Jpn Acad Ser B Phys Biol Sci. 2011;87:152–66. Available from: https://www.ncbi.nlm.nih.gov/pmc/articles/PMC3149377/pdf/pjab-87-152.pdf
5. Puccioni-Sohler M, Grassi MFR, Galvão-Castro B, Caterino A, Proietti AB, Vicente ACP, et al. Increasing awareness of human T-lymphotropic virus type-1 infection: a serious, invisible, and neglected health problem in Brazil. Rev Soc Bras Med Trop. 2019;52:e20190343. 10.1590/0037-8682-0343-2019
